# SCRUM-Japan MONSTAR3 hematology cohort: a nationwide multi-omics integrated platform for next-generation precision medicine in hematologic malignancies

**DOI:** 10.1007/s10147-026-03049-4

**Published:** 2026-05-23

**Authors:** Kensuke Matsuda, Junichiro Yuda, Ryo Yoshimaru, Isamu Harima, Hajime Sakuma, Atsushi Uehara, Masafumi Oto, Masahiko Fukatsu, Takayuki Ikezoe, Hiroaki Araie, Naoko Hosono, Chikako Ohwada, Chiaki Nakaseko, Seiji Kakiuchi, Takao Fujisawa, Tadayoshi Hashimoto, Taro Shibuki, Mitsuho Imai, Michiko Nagamine, Shingo Sakashita, Riu Yamashita, Akio Dodo, Satoshi Horasawa, Yoshiaki Nakamura, Hideaki Bando, Takayuki Yoshino

**Affiliations:** 1 Department of Hematology, National Cancer Center Hospital East, Kashiwa, Japan; 2 Division of Cancer Immunology, Research Institute/Exploratory Oncology Research and Clinical Trial Center (EPOC), National Cancer Center, Kashiwa, Japan; 3 Department of Hematology and Medical Oncology, Japanese Red Cross Kumamoto Hospital, Kumamoto, Japan; 4 Department of Hematology, Yodogawa Christian Hospital, Osaka, Japan; 5 Division of Hematology/Oncology, Department of Internal Medicine, Kameda Medical Center, Kamogawa, Japan; 6 Department of Hematology, Fukushima Medical University Hospital, Fukushima, Japan; 7 Department of Hematology and Oncology, University of Fukui, Fukui, Japan; 8 Department of Hematology, International University of Health and Welfare, Narita, Japan; 9 Translational Research Support Office, National Cancer Center Hospital East, Kashiwa, Japan; 10 Department of Head and Neck Medical Oncology, National Cancer Center Hospital East, Kashiwa, Japan; 11 Department of Gastroenterology and Gastrointestinal Oncology, National Cancer Center Hospital East, Kashiwa, Japan; 12 Department of Hepatobiliary and Pancreatic Oncology, National Cancer Center Hospital East, Kashiwa, Japan; 13 Department of Pathology and Clinical Laboratories, National Cancer Center Hospital East, Kashiwa, Japan; 14 Division of Pathology, Exploratory Oncology Research and Clinical Trial Center, National Cancer Center, Kashiwa, Japan; 15 Division of Translational Informatics, Exploratory Oncology Research and Clinical Trial Center, National Cancer Center Hospital East, Kashiwa, Japan

**Keywords:** SCRUM-Japan, MONSTAR3, Multi-omics profiling, Measurable residual disease, Precision medicine, Spatial transcriptomics

## Abstract

**Background:**

Hematologic malignancies exhibit marked biological heterogeneity that is often insufficiently characterized by genomic profiling alone. Integrated multi-omics approaches are required to enable more accurate prognostic stratification, elucidate resistance mechanisms, and identify therapeutic vulnerabilities across lymphoma, leukemia, and plasma cell neoplasms.

**Methods:**

SCRUM-Japan MONSTAR3 is a nationwide, prospective, integrated multi-omics platform. The hematology cohort aims to enroll 400 patients with newly diagnosed or relapsed/refractory hematologic malignancies. Tumor specimens—including bone marrow aspirates/biopsies or lymph node tissues—are collected at diagnosis and at relapse. The multi-omics workflow encompasses whole-exome sequencing, whole-transcriptome sequencing, spatial transcriptomics, plasma proteomics, metabolomics, microbiome analysis, and tumor-informed measurable residual disease (MRD) monitoring. MRD is assessed using next-generation sequencing-based immunoglobulin heavy (IgH) and T-cell receptor (TCR) rearrangement analysis for lymphoid malignancies and whole-genome sequencing–based variant tracking for myeloid malignancies.

**Results:**

Patient enrollment began in December 2024, followed by nationwide multicenter activation in November 2025. Multi-omics analyses have been implemented in a stepwise manner. Early operational indicators, including biospecimen acquisition, data quality control, and initiation of molecular assays, demonstrate the feasibility of coordinated nationwide deployment of this complex platform.

**Conclusion:**

The MONSTAR3 hematology cohort represents the first nationwide integrated multi-omics initiative dedicated to hematologic malignancies. Its large scale, standardized biospecimen framework, and capacity to incorporate emerging technologies provide a robust infrastructure for molecular stratification, longitudinal disease monitoring, and hypothesis-driven interventional research, thereby advancing clinically actionable precision hematology.

## Introduction

Over the past decade, precision oncology has established the clinical utility of DNA- and RNA-based molecular profiling for identifying actionable genomic alterations and guiding biomarker-driven therapies. However, in hematologic malignancies—many of which are rare and biologically diverse—targeted genomic profiling alone frequently provides limited prognostic and therapeutic guidance throughout the disease course. This limitation is particularly evident in lymphoid neoplasms, where genomic information often fails to reliably predict clinical outcomes or inform optimal treatment selection [1, 2]. Accumulating evidence indicates that this shortcoming reflects the intrinsic biological complexity of hematologic malignancies, in which interactions between malignant cells, the immune system, and the tumor microenvironment play pivotal roles in disease progression, treatment resistance, and relapse [2, 3]. These critical determinants of clinical behavior are not adequately captured by conventional DNA- or RNA-based sequencing approaches alone.

To address these challenges, integrated multi-omics strategies that interrogate tumor-intrinsic molecular alterations alongside spatially resolved microenvironmental and immune features have emerged as a promising approach. Such comprehensive profiling can improve molecular classification, uncover mechanisms of therapeutic resistance, and enable longitudinal monitoring of disease dynamics. Despite these advantages, the implementation of large-scale multi-omics platforms in hematologic malignancies remains challenging. Low disease incidence, variability in diagnostic practices, and heterogeneous treatment paradigms complicate standardized biospecimen collection, assay harmonization, and integrated data analysis. Consequently, nationwide precision hematology initiatives remain limited.

The SCRUM-Japan MONSTAR-SCREEN 3 (MONSTAR3) program was established to overcome these barriers by providing a harmonized, nationwide infrastructure for integrated molecular profiling across both solid tumors and hematologic malignancies. SCRUM-Japan, one of the world’s largest precision medicine networks, has previously demonstrated the feasibility of large-scale patient enrollment and the generation of clinically meaningful evidence in solid tumors. Building on this foundation, MONSTAR3 expands beyond genomic screening to a comprehensive multi-omics ecosystem and extends its scope to hematologic malignancies [4–8].

Here, we describe the design, implementation, and early operational feasibility of the hematology cohort within the MONSTAR3 program.

### Objectives

The primary objective of this study is to establish and characterize a nationwide, integrated multi-omics platform for hematologic malignancies and to evaluate its feasibility for clinical application. Specifically, the platform integrates comprehensive tumor and microenvironment profiling with tumor-informed measurable residual disease (MRD) monitoring and longitudinal plasma-based assays to support molecular stratification, dynamic risk assessment, and efficient enrollment into biomarker-driven clinical trials.

## Patients and methods

### Study design and setting

The MONSTAR3 hematology cohort is conducted within the framework of SCRUM-Japan MONSTAR-SCREEN 3 (MONSTAR3), a nationwide, prospective, multi-omics precision medicine platform that aims to enroll approximately 3200 patients overall, including about 400 patients with hematologic malignancies. The program is led by the National Cancer Center Hospital East and implemented through a nationwide network of participating academic institutions in collaboration with industry partners.

All molecular analyses are performed in qualified laboratories using harmonized, standardized protocols. Centralized governance structures ensure consistency in data quality assurance, ethical oversight, and privacy protection across participating sites. Patient enrollment is ongoing nationwide.

Within the hematology cohort, tumor specimens are collected at diagnosis and at relapse. Bone marrow aspirates or biopsy samples are obtained from patients with leukemia or plasma cell neoplasms, whereas lymph node biopsy specimens are collected from patients with lymphoma. Peripheral blood samples are obtained at diagnosis, at completion of therapy, and at relapse. Stool samples for microbiome analysis are collected at diagnosis, at completion of therapy, six months after treatment completion, and at relapse.

**Fig. 1:**
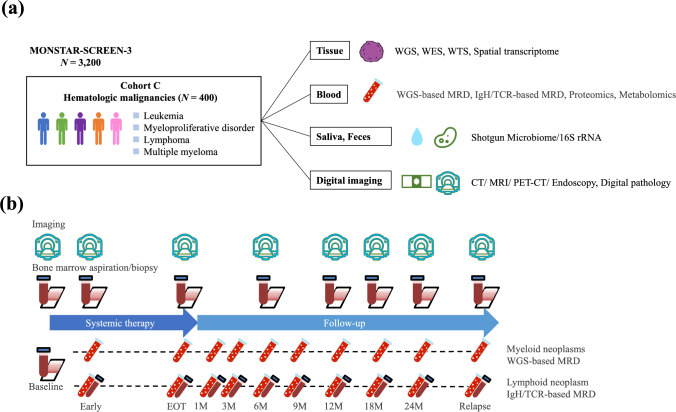
Overview of the MONSTAR3 Hematology Cohort. **a** Schematic overview of the multi-omics analytic framework implemented in the MONSTAR3 hematology cohort, including integrated genomic, transcriptomic, proteomic, and spatial profiling workflows applied to patient biospecimens. **b** Schedule and methodology of measurable residual disease (MRD) monitoring, illustrating longitudinal sample collection and whole-genome sequencing–based tumor-informed MRD assessment throughout the treatment course

MRD monitoring is conducted at predefined time points, including baseline (diagnosis), during treatment, at completion of therapy, at 1, 3, 6, 9, 12, 18, and 24 months after treatment completion, and at relapse. An overview of the assay workflow and MRD monitoring schedule is provided in Fig. 1a, b, respectively.

### Study participants

The study aims to enroll 400 adult patients (≥ 18 years) with newly diagnosed or relapsed/refractory hematologic malignancies who are scheduled to initiate systemic anticancer therapy at a participating institution. Eligibility requires a pathologically or cytologically confirmed diagnosis of a hematologic malignancy, including acute leukemia, myelodysplastic syndrome, lymphoma, plasma cell neoplasm, or myeloproliferative neoplasm.

Key exclusion criteria include the presence of a concurrent or previously diagnosed uncontrolled malignancy within 3 years prior to enrollment; severe or uncontrolled intercurrent illness that could compromise patient safety or protocol compliance; and any condition that, in the judgment of the investigator, would preclude study participation or the safe acquisition of required biospecimens.

### Assays: whole-exome and whole-transcriptome sequencing

Current clinical genomic profiling relies primarily on DNA-targeted sequencing, which often provides insufficient information for accurate prognostication and treatment selection. Limitations include suboptimal detection of fusion genes and splice-site variants using DNA-only panels as well as the inability to assess biomarkers whose clinical relevance depends on RNA or protein expression [9].

To address these limitations, integrated whole-exome sequencing (WES) and whole-transcriptome sequencing (WTS) are performed. DNA and RNA extracted from diagnostic tumor specimens are analyzed at a College of American Pathologists–accredited laboratory (GxD Inc., Kashiwa, Japan) using harmonized analytical workflows.

WES enables comprehensive variant detection and biomarker assessment, including microsatellite instability, tumor mutational burden, and loss of heterozygosity. The assay covers the entire exome at a mean on-target depth exceeding 300 × , with ultra-deep sequencing (> 1000 ×) applied to a clinically curated panel of approximately 700 genes. This design allows reliable detection of single-nucleotide variants, insertions/deletions, and splice-site variants down to an analytically validated variant allele fraction of approximately 0.1–1%.

WTS generates more than 40 million paired-end reads per sample, supporting robust gene fusion detection and transcriptome-wide expression profiling.

### Assays: tumor-informed MRD monitoring

MRD has emerged as a validated endpoint for high-resolution risk stratification and early relapse prediction in hematologic malignancies [10–14]. As a quantitative indicator of remission depth beyond conventional morphologic assessment, MRD serves as a dynamic biomarker throughout the disease course. Accordingly, the MONSTAR3 hematology cohort prospectively evaluates MRD on a nationwide scale.

In lymphoid malignancies, MRD assessment based on immunoglobulin heavy chain (IgH) and T-cell receptor (TCR) rearrangements is well established; however, cross-disease standardization remains limited, and robust MRD approaches for myeloid neoplasms lacking recurrent targetable mutations are scarce [15–17]. To address these challenges, the MONSTAR3 platform implements pan-hematologic, tumor-informed MRD strategies.

For lymphoid malignancies, next-generation sequencing (NGS)–based MRD monitoring is performed in collaboration with GxD (Kashiwa, Japan). Patient-specific IgH/TCR rearrangements are identified at baseline, and individualized sequencing panels are constructed for longitudinal MRD quantification. Although NGS-based IgH/TCR MRD assessment provides one to two orders of magnitude greater sensitivity than multiparametric flow cytometry and yields clinically actionable information, it remains unapproved for routine clinical use in Japan. The clonoSEQ assay, which employs multiplex PCR followed by NGS to identify and track clonotypic IgH/TCR rearrangements, has demonstrated robust analytical validity in acute lymphoblastic leukemia, chronic lymphocytic leukemia, and multiple myeloma and serves as a conceptual reference for the MONSTAR3 MRD framework [18]. Here, the LymphoTrack assay—a clonoSEQ-like multiplex PCR–based NGS approach—is prospectively evaluated in Japanese patients to generate clinical and analytical evidence supporting potential future regulatory approval.

For myeloid malignancies, whole-genome sequencing (WGS) is performed in collaboration with Myriad Genetics Inc. (Salt Lake City, Utah) to define individualized tumor-specific variant sets. These variants are subsequently used for high-sensitivity circulating tumor DNA (ctDNA) detection in serial plasma samples. WGS-based MRD monitoring represents a key innovation of this platform, as it enables tumor-informed tracking without reliance on predefined actionable mutations, an important advantage for myeloid neoplasms characterized by heterogeneous mutational landscapes.

### Assays: spatial transcriptomics

Tumor tissues in hematologic malignancies exhibit marked cellular heterogeneity, comprising malignant cells, stromal components, and diverse populations of tumor-infiltrating immune cells. Conventional bulk profiling approaches cannot resolve this complexity at high spatial resolution. Given the widespread use of immune-based therapies—such as chimeric antigen receptor T-cell (CAR-T) therapies and bispecific antibodies—spatial omics approaches are particularly valuable for dissecting tumor–stroma–immune interactions [19–21].

The spatial organization of the bone marrow and lymph node microenvironments plays a critical role in therapeutic response and resistance [22]. Recent spatial transcriptomic studies have characterized the bone marrow niche in multiple myeloma, identifying spatially distinct gene expression programs and immune gradients associated with disease progression and therapeutic response [23, 24]. Similarly, in diffuse large B-cell lymphoma, spatial profiling has revealed discrete cellular niches with distinct patterns of T-cell activation and exhaustion, thereby highlighting potential therapeutic targets [22].

In the MONSTAR3 study, spatial transcriptomic profiling is performed at diagnosis and relapse across all hematologic malignancy subtypes. RNA in situ hybridization panels enable high-resolution mapping of cellular states, lineage plasticity, immune niches, and the spatial distribution of immune checkpoint ligands. Analytical targets include tumor-associated antigens, markers of T- and natural killer–cell exhaustion, antigen-presentation pathways, macrophage-related programs, and stromal or vascular markers. By systematically characterizing the tumor microenvironment in bone marrow specimens (for leukemias and plasma cell neoplasms) and lymph node specimens (for lymphomas), this platform aims to identify spatial determinants of response and resistance to advanced immunotherapies, including CAR-T cell and bispecific antibody treatments.

Formalin-fixed, paraffin-embedded specimens are analyzed at GxD (Kashiwa, Japan) using the Xenium In Situ platform (10x Genomics) in accordance with manufacturer-recommended protocols. In addition to commercially available panels, GxD develops cancer-focused custom probe sets derived from curated single-cell transcriptomic reference datasets, with probe specificity and on-target performance analytically validated prior to use. Imaging data are processed using vendor-provided pipelines to generate cell-level and region-level expression matrices for downstream analyses of tumor microenvironment architecture and functional states.

### Assays: plasma proteomics and metabolomics

To identify therapeutic targets and biomarkers not captured by genomic profiling, plasma-based proteomic and metabolomic analyses are conducted. Although accumulating evidence implicates protein- and metabolite-level dysregulation in the pathogenesis and progression of hematologic malignancies, comprehensive profiling across disease subtypes remains limited.

Plasma proteomic profiling is performed using the Olink proximity extension assay (PEA) platform (Olink Proteomics AB, Uppsala, Sweden). In this assay, each target protein is recognized by a matched antibody pair conjugated to unique oligonucleotides; dual binding brings the oligonucleotides into proximity, enabling enzymatic extension and generation of a DNA reporter that is subsequently quantified by NGS as a surrogate measure of protein abundance [25]. This dual-recognition, nucleic acid–based readout provides high analytical sensitivity (sub-ng/mL) from low plasma input volumes. High-throughput profiling using the Olink Explore HT platform is performed and analyzed at GxD (Kashiwa, Japan).

This approach enables systematic evaluation of disease-relevant circulating proteins in B-cell malignancies, including B-cell maturation antigen (BCMA), B-cell activating factor (BAFF), and a proliferation-inducing ligand (APRIL), which are implicated in plasma cell and B-cell survival pathways and are actively explored as therapeutic targets in multiple myeloma and related disorders [26]. Soluble BCMA has been reported to correlate with tumor burden and treatment response and may also attenuate the efficacy of BCMA-directed therapies by acting as an antigen sink in settings of high disease burden [27, 28]. In addition, during treatment with emerging immune effector cell therapies such as CAR-T cells and bispecific antibodies, comprehensive proteomic profiling of cytokines and immune mediators may refine characterization of therapeutic responses and immune-related adverse events, including cytokine release syndrome and immune effector cell–associated neurotoxicity syndrome.

Metabolomic analyses are conducted using nuclear magnetic resonance spectroscopy at the Tohoku Medical Megabank Organization (Sendai, Japan). Although generally less sensitive than mass spectrometry, NMR-based metabolomics provides a robust and highly reproducible quantitative workflow suitable for large-scale metabolic profiling [29].

### Assays: microbiome analysis

Emerging evidence indicates that gut microbiome dysbiosis modulates tumor immunity and influences responses to anticancer therapies [30–32]. In this hematology cohort, longitudinal stool sampling is integrated with multi-omics tumor profiling and detailed clinical outcome data. Microbiome sequencing and bioinformatic analyses are performed by Miyarisan Pharmaceutical Co., Ltd. (Tokyo, Japan). Given the increasing use of immune-based therapies in hematologic malignancies, including CAR-T cell and bispecific antibody treatments, this study design enables systematic evaluation of microbiome–treatment response relationships and the identification of microbiome-associated biomarkers.

### Data management, governance, and ethics

The MONSTAR3 program employs a centralized electronic data capture system with role-based access control and comprehensive audit trails to integrate clinical data, imaging data, and multi-omics datasets. For the hematology cohort, additional disease-specific variables—including bone marrow examination findings, flow cytometry results, and cytogenetic analyses (G-banding and fluorescence in situ hybridization)—are systematically collected. Integration of these multimodal datasets enables data-driven analyses across hematologic disease subtypes.

The study is conducted in accordance with the Declaration of Helsinki and applicable national regulations. Institutional review board approval was obtained at each participating institution, and written informed consent was obtained from all participants. Data sharing and secondary use are governed by program-level policies established for the SCRUM-Japan/MONSTAR3 initiative (UMIN000053975).

## Results

### Current status

Patient enrollment in the MONSTAR3 hematology cohort is currently ongoing across participating institutions. The first patient was enrolled at the National Cancer Center Hospital East in December 2024, followed by nationwide multicenter activation in November 2025. As of February 13, 2026, a total of 71 patients had been registered, comprising 46 patients with lymphoid malignancies and 25 with myeloid malignancies.

Enrolled patients have received a broad spectrum of contemporary systemic therapies, including antibody–drug conjugates, bispecific antibodies, and other molecularly targeted or immune-based agents. Multi-omics workflows have been introduced in a stepwise manner, with feasibility assessments integrated during the early phases of implementation. Key operational milestones—such as successful biospecimen acquisition, timely initiation of molecular assays, and execution of integrated data-processing pipelines—have been consistently achieved. These outcomes collectively demonstrate the feasibility of coordinated, nationwide deployment of a comprehensive multi-omics platform for hematologic malignancies.

In accordance with the predefined study objectives, analyses of treatment efficacy and clinical outcomes are not reported at this stage. The present manuscript focuses exclusively on the establishment of the platform and evaluation of its operational feasibility.

## Discussion

### Integrated multi-omics approaches in hematologic malignancies

Integrated multi-omics approaches have emerged as powerful tools for addressing the inherent limitations of conventional clinical genomic profiling in hematologic malignancies. Accumulating evidence indicates that comprehensive molecular characterization—spanning genomic, transcriptomic, proteomic, and metabolomic layers—can uncover biologically and clinically relevant disease subtypes, prognostic biomarkers, and therapeutic vulnerabilities that remain undetected by single-modality analyses [33]. For example, in melanoma, a recent prospective study demonstrated that integrated multi-omics tumor profiling can provide clinically useful information to support treatment decision-making in a molecular tumor board setting [34]. These approaches are particularly well suited to hematologic malignancies, in which disease behavior is governed not only by tumor-intrinsic genomic alterations but also by dynamic interactions with the surrounding cellular and immune microenvironment.

Among recent technological advances, spatial transcriptomics has provided unprecedented resolution in the analysis of tumor–microenvironment interactions. In large B-cell lymphoma, spatial profiling has identified discrete cellular niches defined by divergent patterns of T-cell activation and exhaustion, thereby revealing potentially targetable inflammatory microenvironments [22]. Similarly, in multiple myeloma, spatial transcriptomic studies have elucidated the architectural organization of the bone marrow niche, identifying spatially segregated gene expression programs associated with disease pathogenesis, including pathways related to NETosis and interleukin-17 signaling. Notably, these analyses have demonstrated that T-cell exhaustion is spatially correlated with proximity to malignant plasma cells [23, 24]. Collectively, these findings highlight the critical role of spatial context in shaping disease progression and therapeutic response.

WGS is also gaining increasing clinical relevance, particularly in acute myeloid leukemia, where comprehensive detection of structural variants, copy number alterations, and single-nucleotide variants can directly inform diagnostic classification and risk stratification. The ChromoSeq approach, a streamlined WGS assay, has demonstrated the capacity to detect all clinically relevant genomic events within clinically actionable turnaround times, leading to reclassification of risk in a substantial proportion of patients [35]. Ongoing multicenter studies continue to validate WGS as a precision diagnostic tool [36, 37].

Within this evolving diagnostic landscape, the WGS–based minimal residual disease monitoring strategy implemented in MONSTAR3 represents a conceptual extension of baseline genomic profiling toward longitudinal disease surveillance. By enabling tumor-informed tracking without dependence on predefined targetable mutations, this strategy provides a flexible framework for refining therapeutic decision-making, particularly in myeloid malignancies. In lymphoid neoplasms, molecular and genomic profiling is similarly becoming central to therapeutic selection, with biomarker-driven clinical trials increasingly guiding the application of CAR-T cell therapies, bispecific antibodies, and molecularly targeted agents [38].

Collectively, these advances position MONSTAR3 as a next-generation precision oncology platform that integrates advanced multi-omics technologies to facilitate deeper biological insight, more accurate prognostication, and increasingly informed therapeutic decision-making in hematologic malignancies.

### Strength and future perspectives

The MONSTAR3 hematology cohort establishes a nationwide, highly coordinated research infrastructure that integrates standardized biospecimen logistics with state-of-the-art multi-omics profiling. Previous large-scale precision oncology initiatives, such as NCI-MATCH, have demonstrated the clinical and translational value of genomics-driven decision frameworks [39]. These approaches largely focus on genomic alterations within biomarker-driven clinical trial frameworks. In contrast, the MONSTAR3 hematology cohort, built on the SCRUM-Japan platform, integrates longitudinal multi-omics profiling—including genomics, transcriptomics, spatial transcriptomics, proteomics, metabolomics, microbiome analysis, and MRD monitoring—within an open nationwide clinical research network designed for continuous patient enrollment, broad institutional participation, and collaboration with industry-sponsored clinical trials.

This structure enables systematic accumulation of harmonized multi-omics and clinical data across a wide spectrum of hematologic malignancies. As a platform dedicated specifically to hematologic malignancies, MONSTAR3 offers distinct advantages in scale, disease diversity, and analytical depth, thereby supporting translational research that is closely aligned with real-world clinical practice. Importantly, the program is explicitly designed to address challenges inherent to hematologic malignancies—including disease rarity, therapeutic heterogeneity, and variability in diagnostic workflows—through standardized data collection and integrated analytical pipelines. The scale and complexity of these integrated datasets necessitate advanced computational approaches for effective analysis and interpretation. To enable artificial intelligence (AI)-driven integration of multi-omics data, we have established a high-performance computing system named VAPOR CONE [8]. Using this VAPOR CONE platform, we are currently developing AI-based classifiers that integrate multi-omics data, including genomic, proteomic, and other molecular features, to detect cancer and predict the tumor tissue of origin. These classifiers will guide the development of innovative MRD and multi-cancer early detection (MCED) assays [40].

Beyond its scientific contributions, the MONSTAR3 platform has important implications for global drug development. In hematology, regulatory approvals in the United States have frequently been based on early-phase clinical trials, reflecting disease rarity and often limiting patient participation outside North America and Europe [41]. The expanding role of emerging biotechnology companies, many of which lack extensive international trial networks, has further exacerbated these disparities [42]. As a result, regional delays in drug approval have become an increasingly recognized challenge in hematologic oncology. In this context, a scalable, trial-ready infrastructure capable of rapid patient identification and enrollment is essential. By providing such a framework, MONSTAR3 has the potential not only to generate high-resolution biological insights but also to enhance access to clinical trials and reduce geographic inequities in the development and delivery of novel therapies.

In summary, MONSTAR3 represents a foundational step toward a more precise, coordinated, and globally inclusive paradigm for the study and treatment of hematologic malignancies.

## References

[1] Fukuhara S, Oshikawa-Kumade Y, Kogure Y et al (2022) Feasibility and clinical utility of comprehensive genomic profiling of hematological malignancies. Cancer Sci 113:2763–2777. 10.1111/cas.15427 35579198 10.1111/cas.15427 PMC9357666

[2] de Leval L, Alizadeh AA, Bergsagel PL et al (2022) Genomic profiling for clinical decision making in lymphoid neoplasms. Blood 140:2193–2227. 10.1182/blood.2022015854 36001803 10.1182/blood.2022015854 PMC9837456

[3] Miyawaki K, Sugio T (2022) Lymphoma microenvironment in DLBCL and PTCL-NOS: the key to uncovering heterogeneity and the potential for stratification. J Clin Exp Hematop 62:127–135. 10.3960/jslrt.22027 36171096 10.3960/jslrt.22027 PMC9635031

[4] Nakamura Y, Fujisawa T, Taniguchi H et al (2021) SCRUM-Japan GI-SCREEN and MONSTAR-SCREEN: path to the realization of biomarker-guided precision oncology in advanced solid tumors. Cancer Sci 112:4425–4432. 10.1111/cas.15132 34510657 10.1111/cas.15132 PMC8586659

[5] Nakamura Y, Taniguchi H, Ikeda M et al (2020) Clinical utility of circulating tumor DNA sequencing in advanced gastrointestinal cancer: SCRUM-Japan GI-SCREEN and GOZILA studies. Nat Med 26:1859–1864. 10.1038/s41591-020-1063-5 33020649 10.1038/s41591-020-1063-5

[6] Aoki Y, Nakamura Y, Denda T et al (2023) Clinical validation of plasma-based genotyping for RAS and BRAF V600E mutation in metastatic colorectal cancer: SCRUM-Japan GOZILA substudy. JCO Precis Oncol 7:e2200688. 10.1200/po.22.00688 37343204 10.1200/PO.22.00688 PMC10309506

[7] Taniguchi H, Nakamura Y, Kotani D et al (2021) CIRCULATE-Japan: circulating tumor DNA-guided adaptive platform trials to refine adjuvant therapy for colorectal cancer. Cancer Sci 112:2915–2920. 10.1111/cas.14926 33931919 10.1111/cas.14926 PMC8253296

[8] Hashimoto T, Nakamura Y, Fujisawa T et al (2024) The SCRUM-MONSTAR cancer-omics ecosystem: striving for a quantum leap in precision medicine. Cancer Discov 14:2243–2261. 10.1158/2159-8290.cd-24-0206 39023403 10.1158/2159-8290.CD-24-0206 PMC11528206

[9] Owen D, Ben-Shachar R, Feliciano J et al (2024) Actionable structural variant detection via RNA-NGS and DNA-NGS in patients with advanced non-small cell lung cancer. JAMA Netw Open 7:e2442970. 10.1001/jamanetworkopen.2024.42970 39495511 10.1001/jamanetworkopen.2024.42970 PMC11536281

[10] Kurtz DM, Scherer F, Jin MC et al (2018) Circulating tumor DNA measurements as early outcome predictors in diffuse large B-cell lymphoma. J Clin Oncol 36:2845–2853. 10.1200/jco.2018.78.5246 30125215 10.1200/JCO.2018.78.5246 PMC6161832

[11] Berry DA, Zhou S, Higley H et al (2017) Association of minimal residual disease with clinical outcome in pediatric and adult acute lymphoblastic leukemia. JAMA Oncol 3:e170580. 10.1001/jamaoncol.2017.0580 28494052 10.1001/jamaoncol.2017.0580 PMC5824235

[12] van Dongen JJM, van der Velden VHJ, Brüggemann M et al (2015) Minimal residual disease diagnostics in acute lymphoblastic leukemia: need for sensitive, fast, and standardized technologies. Blood 125:3996–4009. 10.1182/blood-2015-03-580027 25999452 10.1182/blood-2015-03-580027 PMC4490298

[13] Perrot A, Lauwers-Cances V, Corre J et al (2018) Minimal residual disease negativity using deep sequencing is a major prognostic factor in multiple myeloma. Blood 132:2456–2464. 10.1182/blood-2018-06-858613 30249784 10.1182/blood-2018-06-858613 PMC6284215

[14] Scherer F, Kurtz DM, Newman AM et al (2016) Distinct biological subtypes and patterns of genome evolution in lymphoma revealed by circulating tumor DNA. Sci Transl Med 8:364–155. 10.1126/scitranslmed.aai8545 27831904 10.1126/scitranslmed.aai8545 PMC5490494

[15] Logan AC, Gao H, Wang C et al (2011) High-throughput VDJ sequencing for quantification of minimal residual disease in chronic lymphocytic leukemia and immune reconstitution assessment. Proc Natl Acad Sci U S A 108:21194–21199. 10.1073/pnas.1118357109 22160699 10.1073/pnas.1118357109 PMC3248502

[16] Jongen-Lavrencic M, Grob T, Hanekamp D et al (2018) Molecular minimal residual disease in acute myeloid leukemia. N Engl J Med 378:1189–1199. 10.1056/nejmoa1716863 29601269 10.1056/NEJMoa1716863

[17] Heuser M, Freeman SD, Ossenkoppele GJ et al (2021) 2021 update on MRD in acute myeloid leukemia: a consensus document from the European LeukemiaNet MRD Working Party. Blood 138:2753–2767. 10.1182/blood.2021013626 34724563 10.1182/blood.2021013626 PMC8718623

[18] Ching T, Duncan ME, Newman-Eerkes T et al (2020) Analytical evaluation of the clonoSEQ Assay for establishing measurable (minimal) residual disease in acute lymphoblastic leukemia, chronic lymphocytic leukemia, and multiple myeloma. BMC Cancer 20:612. 10.1186/s12885-020-07077-9 32605647 10.1186/s12885-020-07077-9 PMC7325652

[19] Matsuda K, Nonami A, Shinohara K et al (2025) Regulatory approval of CAR‐T cell and BsAb products for lymphoid neoplasms in the US, EU, and Japan. Clin Pharmacol Ther 118:118–127. 10.1002/cpt.3645 40084978 10.1002/cpt.3645

[20] Hunter MV, Moncada R, Weiss JM et al (2021) Spatially resolved transcriptomics reveals the architecture of the tumor-microenvironment interface. Nat Commun 12:6278. 10.1038/s41467-021-26614-z 34725363 10.1038/s41467-021-26614-z PMC8560802

[21] Jin Y, Zuo Y, Li G et al (2024) Advances in spatial transcriptomics and its applications in cancer research. Mol Cancer 23:129. 10.1186/s12943-024-02040-9 38902727 10.1186/s12943-024-02040-9 PMC11188176

[22] Dai Y, Kizhakeyil A, Chihara D et al (2025) Multi-modal spatial characterization of tumor immune microenvironments identifies targetable inflammatory niches in diffuse large B cell lymphoma. Nat Genet 57:2715–2727. 10.1038/s41588-025-02353-5 41120574 10.1038/s41588-025-02353-5 PMC12597830

[23] Muiños-Lopez E, Lopez-Perez AR, Sudupe L et al (2025) Characterization of the bone marrow architecture of multiple myeloma using spatial transcriptomics. Commun Biol 8:1620. 10.1038/s42003-025-08975-z 41266582 10.1038/s42003-025-08975-z PMC12634688

[24] de Jong MME, Kellermayer Z, Papazian N et al (2021) The multiple myeloma microenvironment is defined by an inflammatory stromal cell landscape. Nat Immunol 22:769–780. 10.1038/s41590-021-00931-3 34017122 10.1038/s41590-021-00931-3

[25] Assarsson E, Lundberg M, Holmquist G et al (2014) Homogenous 96-plex PEA immunoassay exhibiting high sensitivity, specificity, and excellent scalability. PLoS ONE 9:e95192. 10.1371/journal.pone.0095192 24755770 10.1371/journal.pone.0095192 PMC3995906

[26] Tagami N, Yuda J, Goto Y (2024) Current status of BAFF targeting immunotherapy in b-cell neoplasm. Int J Clin Oncol 29:1676–1683. 10.1007/s10147-024-02611-2 39222149 10.1007/s10147-024-02611-2 PMC11511695

[27] Oura M, Ikeda D, Aikawa S et al (2026) Soluble BCMA as a biomarker reflecting tumor volume and treatment response in Waldenström macroglobulinemia. Leuk Lymphoma 67:85–96. 10.1080/10428194.2025.2569759 41062306 10.1080/10428194.2025.2569759

[28] Costa BA, Ortiz RJ, Lesokhin AM et al (2024) Soluble b‐cell maturation antigen in multiple myeloma. Am J Hematol 99:727–738. 10.1002/ajh.27225 38270277 10.1002/ajh.27225

[29] Emwas AH, Roy R, McKay RT et al (2019) NMR spectroscopy for metabolomics research. Metabolites 9:123. 10.3390/metabo9070123 31252628 10.3390/metabo9070123 PMC6680826

[30] Iida N, Dzutsev A, Stewart CA et al (2013) Commensal bacteria control cancer response to therapy by modulating the tumor microenvironment. Science 342:967–970. 10.1126/science.1240527 24264989 10.1126/science.1240527 PMC6709532

[31] Routy B, Le Chatelier E, Derosa L et al (2018) Gut microbiome influences efficacy of PD-1-based immunotherapy against epithelial tumors. Science 359:91–97. 10.1126/science.aan3706 29097494 10.1126/science.aan3706

[32] Matson V, Fessler J, Bao R et al (2018) The commensal microbiome is associated with anti-PD-1 efficacy in metastatic melanoma patients. Science 359:104–108. 10.1126/science.aao3290 29302014 10.1126/science.aao3290 PMC6707353

[33] Khoury R, Raffoul C, Khater C et al (2025) Precision medicine in hematologic malignancies: evolving concepts and clinical applications. Biomedicines 13:1654. 10.3390/biomedicines13071654 40722725 10.3390/biomedicines13071654 PMC12292076

[34] Miglino N, Toussaint NC, Ring A et al (2025) Feasibility of multiomics tumor profiling for guiding treatment of melanoma. Nat Med 31:2430–2441. 10.1038/s41591-025-03715-6 40425842 10.1038/s41591-025-03715-6 PMC12283375

[35] Duncavage EJ, Schroeder MC, O’Laughlin M et al (2021) Genome sequencing as an alternative to cytogenetic analysis in myeloid cancers. N Engl J Med 384:924–935. 10.1056/NEJMoa2024534 33704937 10.1056/NEJMoa2024534 PMC8130455

[36] Ahmed F, Zhong J (2024) Advances in DNA/RNA sequencing and their applications in acute myeloid leukemia (AML). Int J Mol Sci 26:71. 10.3390/ijms26010071 39795930 10.3390/ijms26010071 PMC11720148

[37] Berglund E, Barbany G, Orsmark-Pietras C et al (2022) A study protocol for validation and implementation of whole-genome and -transcriptome sequencing as a comprehensive precision diagnostic test in acute leukemias. Front Med 9:842507. 10.3389/fmed.2022.842507 10.3389/fmed.2022.842507 PMC8987911 35402448

[38] Nolan J, Kuruvilla J (2025) Diffuse large B-cell lymphoma: What clinical progress have we seen in the last 5 years? Expert Opin Investig Drugs 34:831–853. 10.1080/13543784.2025.2587276 41204727 10.1080/13543784.2025.2587276

[39] O’Dwyer PJ, Gray RJ, Flaherty KT et al (2023) The NCI-MATCH trial: lessons for precision oncology. Nat Med 29:1349–1357. 10.1038/s41591-023-02379-4 37322121 10.1038/s41591-023-02379-4 PMC10612141

[40] Shibuki T, Yamashita R, Hashimoto T et al (2025) Clinical Development of Molecular Residual Disease (MRD) and Multi-Cancer Early Detection (MCED) using Liquid Biopsy Multiomics with Artificial Intelligence (AI). Int J Clin Oncol 20:23. 10.1007/s10147-026-03001-6 10.1007/s10147-026-03001-6 PMC13102821 41790338

[41] Matsuda K, Nagai S, Sugimoto K (2023) Drug approval delays in hematologic malignancies between Europe and the US and between Japan and the US: a clinical perspective. Jpn J Clin Oncol 53:1125–1129. 10.1093/jjco/hyad117 37642224 10.1093/jjco/hyad117

[42] Matsuda K, Nagai S, Sugimoto K (2025) Characteristics of drugs from non-global companies for hematologic malignancies and impact on global regulatory approval. Clin Pharmacol Ther 117:232–239. 10.1002/cpt.3440 39253985 10.1002/cpt.3440 PMC11652803

